# Detection of return of spontaneous circulation during cardiopulmonary resuscitation using continuous carotid artery Doppler blood flow monitored by AI in an animal model

**DOI:** 10.1016/j.resplu.2025.101207

**Published:** 2025-12-24

**Authors:** Raghava Vinaykanth Mushunuri, Bjorn Ove Faldaas, Frank Lindseth, Charlotte Bjork Ingul, Gabriel Kiss

**Affiliations:** a Department of Computer Science (IDI), NTNU, Trondheim, Norway; b Department of Circulation and Medical Imaging (ISB), Norwegian University of Science and Technology, Trondheim, Norway; c Faculty of Nursing and Health Science, Nord University, Bodø, Norway

**Keywords:** Cardiac arrest, Cardio pulmonary resuscitation, Explainable AI, RescueDoppler, Chest compression, Return of spontaneous circulation

## Abstract

**Background:**

Manual pulse checks during cardiopulmonary resuscitation (CPR) to confirm return of spontaneous circulation (ROSC) are often unreliable and time- consuming. To address this, a novel RescueDoppler device has been developed, consisting of a small ultrasound probe that attaches to the neck and continuously monitors potential blood flow in the carotid artery.

**Aim:**

To provide automatic real-time feedback on ROSC using RescueDoppler carotid blood flow during cardiac arrest by employing advanced deep-learning techniques.

**Method:**

We conducted two experiments using carotid blood flow velocity recordings from 9 pigs, with ventricular fibrillation induced via an implantable defibrillator. Experiment 1 included 2610 annotated heart cycles and used a simple classifier to distinguish compression (only manual) from ROSC signals. Experiment 2 involved 5140 cycles and employed a two- stage classifier: the first stage replicated Experiment 1, while the second further separated compression-only from compression with intrinsic cardiac activity. Two- second spectral signals were extracted, normalized, and artificial neural networks are trained for classifying the signals by using State-of-the-art deep learning models as feature extractors. Grad-CAM, an explainable AI (XAI) method, highlighted key regions which contributed most to the model’s predictions.

**Results:**

Our model achieved mean sensitivity of 98 %, specificity of 97 %, positive predictive value of 97 %, and negative predictive value of 100 %. XAI heatmaps highlighted features important for the model’s predictions.

**Conclusion:**

In a porcine model of cardiac arrest, we demonstrated that deep learning techniques can harness the potential of AI to identify the compressions with intrinsic cardiac activity and ROSC during CPR, achieving highly accurate results.

## Introduction

Cardiac arrest affects 4–5 million people annually and ranks as the third leading cause of death worldwide, according to the European Society of Cardiology.^1^ Despite advancements in resuscitation techniques, survival rates remain below 10 % and continue to decrease with each minute that advanced life support is delayed.^2^

Defibrillators automatically detect electrical activity by analyzing electrocardiograms (ECG); however, they lack the essential ability to assess blood flow, which is critical for detecting the return of spontaneous circulation (ROSC). Although manual pulse palpation is standard for assessing circulation, it is often inaccurate and should be limited to 10 s.^3^, ^4^ Continuing cardiopulmonary resuscitation (CPR) unnecessarily can also be harmful.^5^, ^6^ Point-of-care ultrasound (POCUS) provides valuable insights during cardiac arrest but requires pausing compressions and is recommended only for experienced practitioners.^7^, ^8^, ^9^, ^10^

**Fig. 1:**
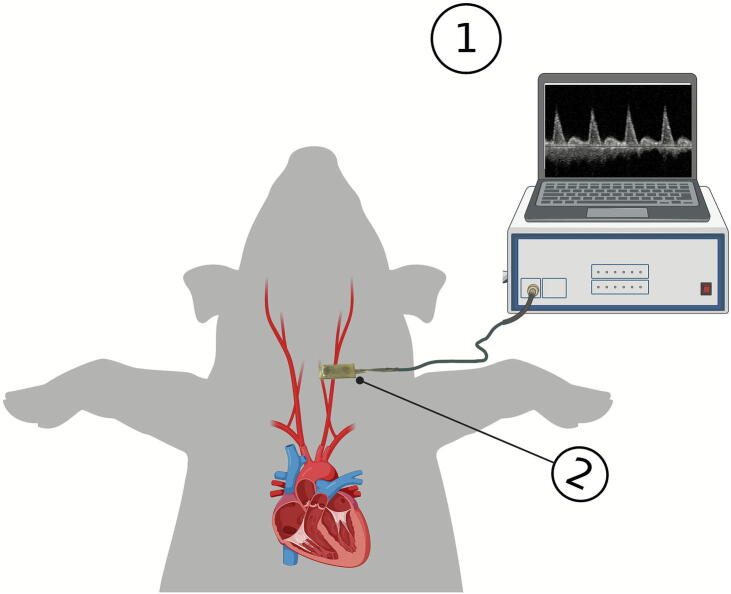
**Illustration of RescueDoppler patch and probe attached over the carotid artery for porcine model. 1. Monitor displaying carotid blood flow velocities, 2. RescueDoppler probe attached to the carotid artery. Figure is made using BioRender support**.

For this purpose, RescueDoppler – a novel hands-free carotid Doppler probe system that continuously measures blood flow velocities in the carotid artery (Fig. 1) –has been developed and tested in preclinical studies by Faldaas et al.^11^ RescueDoppler has shown considerable promise in detecting intrinsic cardiac activity during chest compressions and in identifying ROSC.^11^, ^12^, ^13^, ^14^, ^15^ This state, characterized by a lack of consistent rhythm, is frequently associated with minimal or absent diastolic flow. RescueDoppler was also shown to be feasible for use during resuscitation without interruptions in a clinical pilot study by Krüger et al.^14^, ^15^ By allowing for continuous, non-invasive monitoring, RescueDoppler may enhance the quality of CPR and improve patient outcomes. Since rapid assessment of circulation is crucial during cardiac arrest, integrating Artificial Intelligence (AI) could further support clinical decision-making.

While AI has significantly transformed many medical areas, its application in cardiac arrest management has primarily concentrated on ECG analysis.^16^, ^17^, ^18^, ^19^, ^20^, ^21^ The potential use of AI with carotid Doppler during cardiac arrest remains unexplored but promising. To investigate this opportunity, we developed a deep learning pipeline utilizing retrospective porcine data from Faldaas et al. We employed transfer learning to train five state-of-the-art (SOTA) AI models to distinguish between ROSC signals and those from chest compressions or compressions with intrinsic cardiac activity. Our goal was to enhance the efficiency of RescueDoppler’s performance in emergency care.

## Materials and methods

### Data acquisition

The dataset used in this study comes from recordings of animal experiments conducted by Faldaas et al., where the RescueDoppler system measured carotid artery blood flow velocities during cardiac arrest. This system utilizes a custom-designed pulsed wave Doppler carotid probe featuring two transducers angled at ±30°. The study included recordings from nine pigs (Sus scrofa domesticus), approved by the Norwegian Animal Research Authority (FOTS-ID 25415) and conducted in accordance with ARRIVE guidelines. The group comprised eight male pigs and one female, with an average body weight of 29.8 ± 1.72 kg. Two distinct protocols were used for data collection. In the first protocol, we induced ventricular fibrillation (VF) followed by CPR and shock to achieve ROSC. In the second protocol, VF was induced and immediately terminated by defibrillation to restore cardiac rhythm, after which CPR was performed on a beating heart. Chest compressions were performed manually on seven pigs at rates of 50 or 100 compressions per minute, and mechanically using the LUCAS 3 device on eight pigs.

Manual chest compressions were not immediately followed by mechanical compressions within the same sequence. In total, manual compressions were performed for 1815 s (maximum 375 s, minimum 0 s), and mechanical compressions for 1650 s (maximum 315 s, minimum 0 s), with mean durations of 182 s for manual and 183 s for mechanical compressions. ROSC was achieved in all sequences immediately following defibrillation. Manual compressions lasted between 15 and 30 s, whereas mechanical compressions ranged from 15 to 60 s. VF was induced using the DC Fibber 7 protocol, applying a constant DC voltage of 7.5 V for 2 s from an implantable cardioverter-defibrillator (ICD). Subsequently, another manual DC shock from the ICD was administered to restore sinus rhythm and achieve ROSC. Untreated VF lasted between 5 and 60 s per sequence, with an overall mean duration of 12 s across all animals and sequences. After inducing VF with the initial defibrillation, a subsequent defibrillation intended to restore circulation led to a transitional cardiac state characterized by irregular myocardial activity and intermittent blood flow.

We termed this phenomenon as intrinsic cardiac activity, which is characterized by an irregular heartbeat with antegrade systolic blood flow and no antegrade diastolic blood flow. In the recordings obtained using the second experiment protocol, intrinsic cardiac activity was detected between compressions. Over time, the heart gradually regained adequate positive diastolic flow, ultimately resulting in the achievement of ROSC.

### Data annotation

**Fig. 2:**
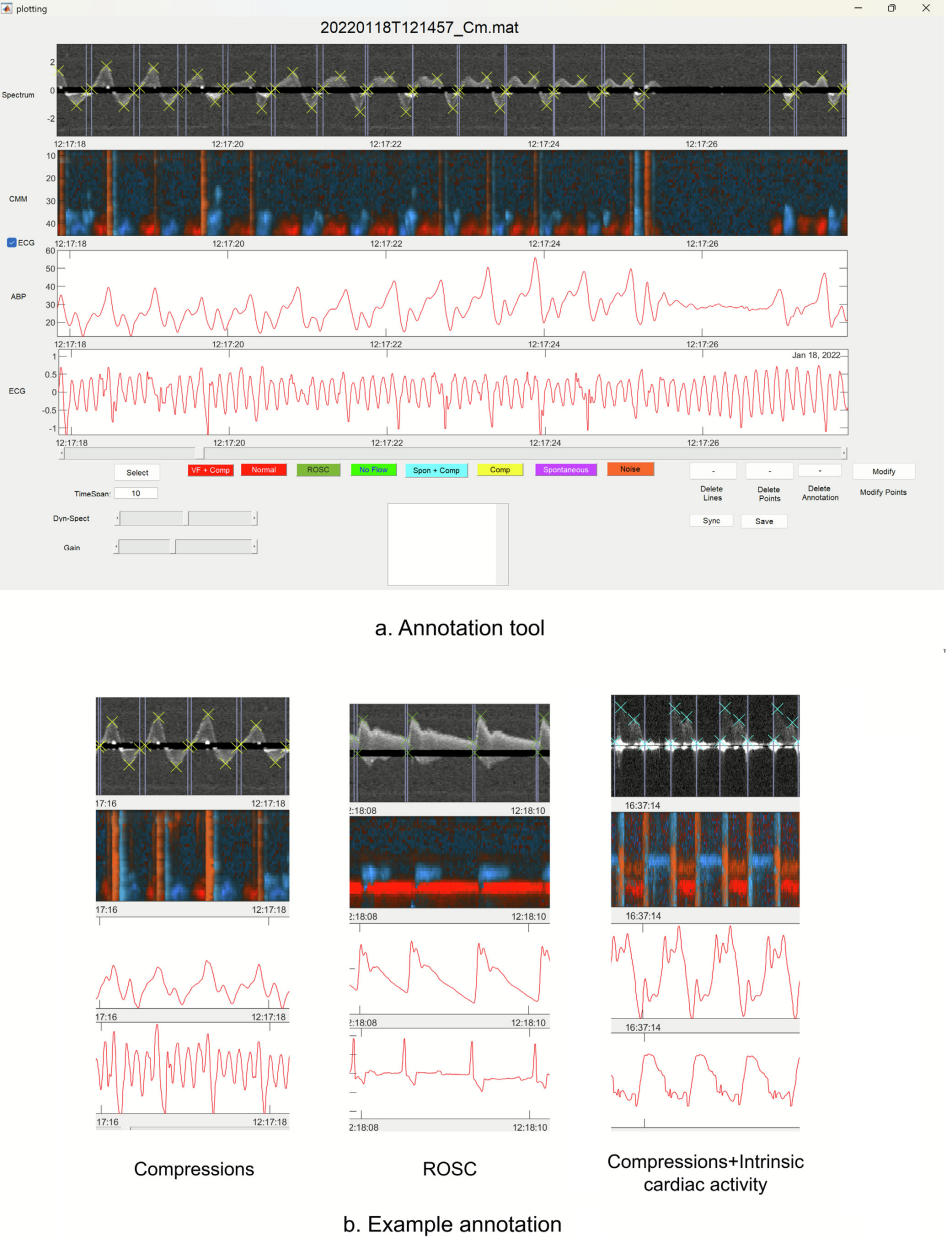
**Illustration of the annotation tool and examples of annotations. Figure a show the annotation tool, and figure b shows example of annotations for each class**.

We developed a MATLAB-based annotation tool (Fig. 2) to label individual heart cycles within the RescueDoppler recordings. Each signal was annotated as one of three categories: chest compressions, compressions with intrinsic cardiac activity, and ROSC. The specific features annotated for each category were as follows:

Chest Compressions: Start of the chest compression cycle, peak positive velocity, peak negative velocity, and end of the chest compression cycle.
Compressions with intrinsic cardiac activity: Start of the cycle, peak positive velocity of the intrinsic cardiac activity and end of intrinsic cardiac activity, peak positive and negative velocities of compressions, and end of the cycle.
ROSC: Start of the heart cycle, peak positive velocity, end-diastole, and end of the cycle.

The structured annotation facilitated precise labeling of hemodynamic patterns, which in turn supported subsequent model training and evaluation. There were two human experts who annotated the 3 categories, a consultant in cardiology and anaesthesiology (CBI) and the first author (RVM). All annotations were checked twice and if any disagreements, CBI had the final decision.

The dataset is divided into two distinct subsets, one for each experiment. For the first experiment, we annotated a total of 2610 heart cycles from five animals. This dataset consisted exclusively of signals from manual chest compressions and without any intrinsic cardiac activity present during the compressions. In the second experiment, 5140 heart cycles from all animal recordings were annotated, including both manual and mechanical compressions. This dataset also contained recordings that included intrinsic cardiac activity signals occurring between the compressions.

### Deep learning-based ROSC detector

**Fig. 3:**
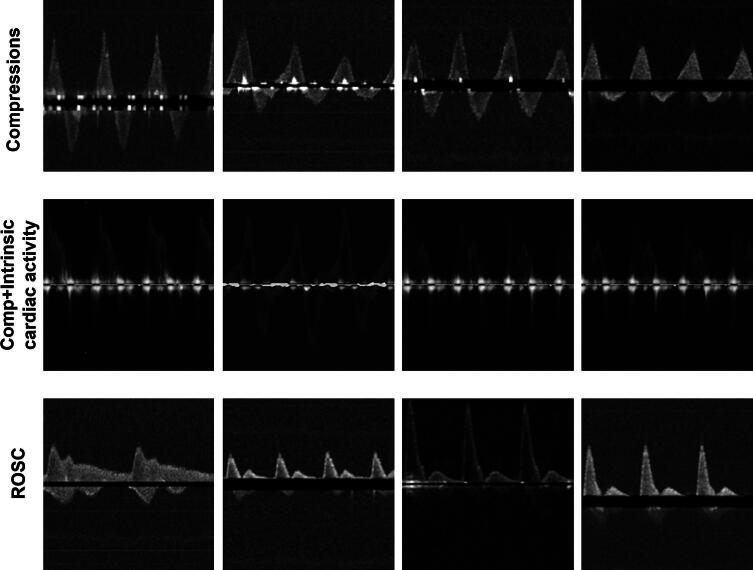
**B-mode spectrum images used for experiments. The first row represents compression signals, the second row represents compressions with intrinsic cardiac activity and the third row represents ROSC signals**.

This study comprises of two experiments, which are designed to carefully evaluate the effectiveness of SOTA models in classifying various blood flow patterns. Five advanced deep learning models – VGG16,^22^ ResNet101,^23^ MobileNet-v3,^24^ EfficientNetv2,^25^ and Data-efficient Image Transformer (DeiT)^26^ – were tested. We selected these models to conduct a broad comparative study across classical and modern convolutional neural network (CNN) models, lightweight architectures, and transformer-based approaches. VGG served as a foundational CNN baseline, while ResNet and EfficientNetv2represent deeper architectures with improved feature propagation. MobileNet was included to assess performance in computationally constrained clinical settings. DeiT enabled evaluation of transformer-based global feature modeling. This comparison allowed us to identify the most suitable architecture for ROSC detection. The first experiment focuses on distinguishing between two principal signal categories: chest compression (manual chest compressions without any intrinsic cardiac activity) and ROSC. For this phase, the input data comprises B-mode signals derived from the spectrum (Fig. 3). The primary objective is to assess whether these models can accurately differentiate between chest compression signals (only manual) and ROSC signals.

In the second experiment, the classification process was refined by distinguishing between signals containing only chest compressions and those that include intrinsic cardiac activity during compressions. For this experiment, we included signals from both manual and mechanical compressions. This distinction was made by introducing a new class label annotation. We implemented a two-stage classifier based on SOTA deep learning models. The first-stage classifier is similar to the one used in Experiment 1. In this stage, signals with intrinsic cardiac activity during compressions are also considered as chest compression signals and are assigned to a single class label, whereas the alternative class label is ROSC.

In the second stage, the same network architecture used in the first experiment was retrained to differentiate between pure chest compression signals and compressions with intrinsic cardiac activity. This stage aimed to refine the classification of various compression signals. During inference, signals initially identified as compressions by the first-stage classifier were forwarded to the second-stage model for more detailed categorization. Although the timing of chest compressions was known during data collection, the model was trained to autonomously detect compressions. The long-term goal is to enable AI-guided feedback, assisting rescuers in delivering high-quality CPR.

**Fig. 4:**
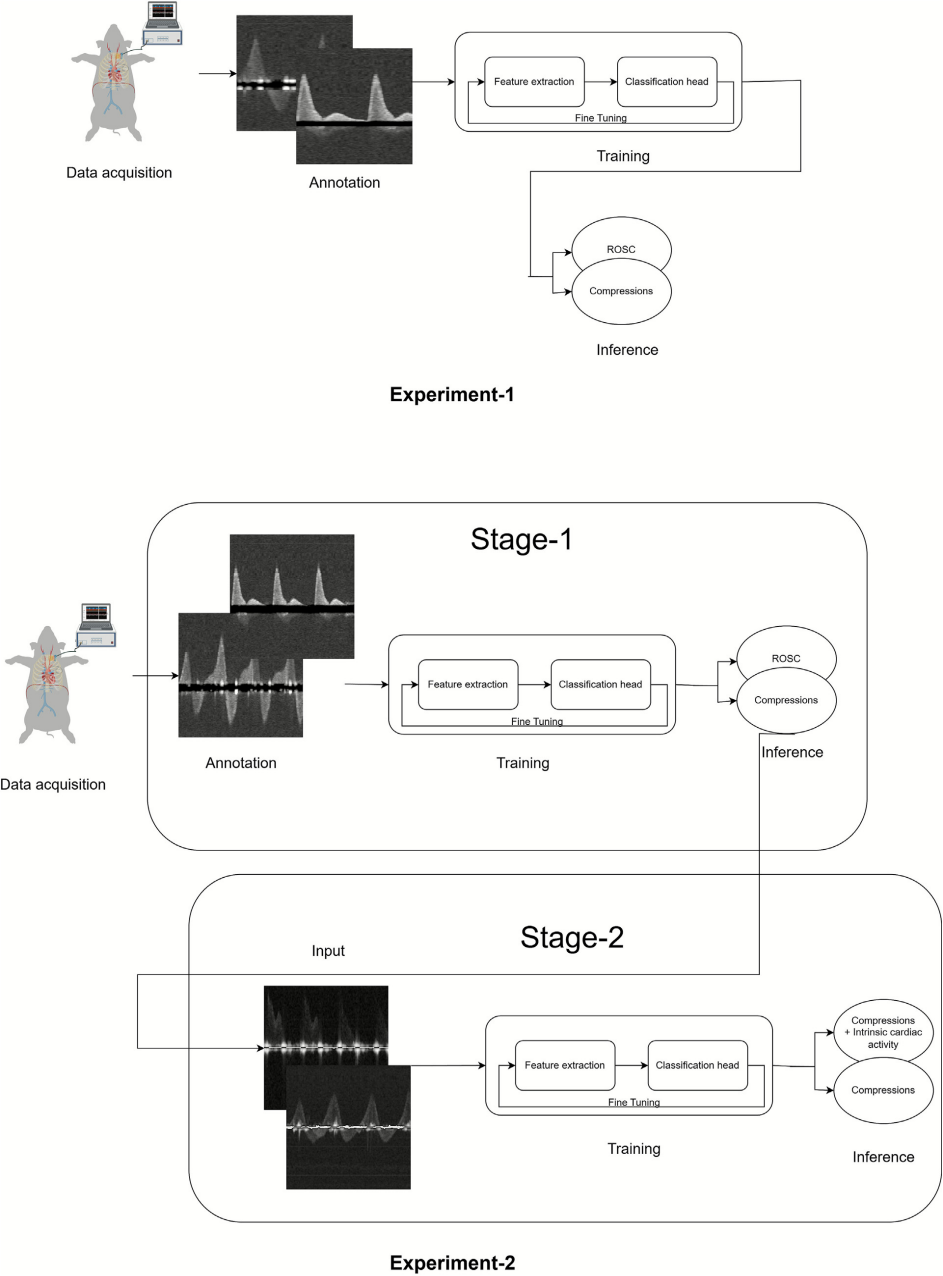
**The pipeline from data acquisition to inference for all the experiments**.

Fig. 4 presents a block diagram depicting the end-to-end pipeline for the individual experiments conducted. This pipeline consists of four key steps: data acquisition, annotation, model training, and inference.

### Training

The networks were trained using the PyTorch framework on an NVIDIA RTX A6000. Model weights, pre-trained on ImageNet,^27^ were obtained from the Torchvision library for initialization. For explainability purposes, we employed the Captum library. Original images with dimensions of 289 × 291 × 1 were reshaped to 224 × 224 × 1 through nearest-neighbor interpolation. A trainable convolution layer was then added to convert these images to 224 × 224 × 3, the format expected by pre-trained models. Two-second pulses were saved as images and were loaded using the PIL (Pillow) library and normalized to a pixel range of 0–1.0. The batch size was set to 32, and a learning rate of 1 × 10^−2^ was implemented. Focal loss was utilized for all experiments, and the ADAM optimizer was applied to optimize model weights. Models underwent training for 200 epochs.

In Experiment 1, we adopted a one-vs-rest strategy and conducted five-fold cross-validation. Experiment 2 utilized leave-one-subject-out two-fold cross-validation, where each iteration designated one subject for testing and the remaining eight for training. Two subjects with annotations for all class labels were selected for the two-fold testing approach.

We utilized transfer learning by starting with ImageNet-pre-trained weights, allowing a model trained on a large dataset to be adapted to a related task. This approach accelerates convergence and enhances performance, particularly when handling limited data, by reusing learned features. In Experiment 1, we initialized the model with pre-trained weights from ImageNet and fine-tuned it on our dataset. The same methodology was applied in Stage 1 of Experiment 2. However, in Stage 2, instead of generic pre-trained weights, we used the model trained in Stage 1 as a fixed feature extractor. The feature extraction layers were frozen, and only the classification head was trained, focusing on distinguishing between pure compression signals and those containing intrinsic cardiac activity.

### Quantitative evaluation

All experiments were evaluated using the one-vs-rest cross-validation method, testing models by excluding data from one animal at a time. This approach ensures robustness and aids in assessing the models’ generalizability across different subjects. We evaluated the model’s performance quantitatively using standard metrics, including sensitivity, specificity, Positive Predictive Value (PPV), Negative Predictive Value (NPV), and overall accuracy (Table 1).

**Table 1:** Evaluation metrics of experiments.

**Network**	**Accuracy %**	**Sensitivity %**	**Specificity %**	**PPV %**	**NPV %**
**Experiment 1**					
VGG16	**94.4**	**94.0**	**95.0**	**95.6**	**93.0**
ResNet101	68.2	63.2	78.2	80.4	68.8
DeiT	58	69	45	69.6	45.4
EfficientNet-V2	66.8	99.6	23.6	65.2	39.4
MobileNet-V3	75.8	64.6	82	66.4	76.8

**Experiment 2**					
VGG16	**98.3**	**98.9**	**97.3**	**97.2**	**100.0**
ResNet101	71.6	68.5	62.6	76.2	62.0
DeiT	84.6	88.9	89.9	87.3	85.5
EfficientNet-V2	62.7	34.7	62.3	33.3	89.2
MobileNet-V3	81.2	88.6	81.2	81.2	81.5

Evaluation metrics for the experiments conducted. In both the experiments, VGG16 outperformed in classifying various classes. In experiment 2 the evaluation metric corresponds to the weighted average from both the stages of the classification model.

### Explainable AI

Explainable AI (XAI) encompasses a set of methods designed to enhance the transparency and interpretability of machine learning models. These techniques clarify how a model reaches its decisions, offering insights into the key features or biomarkers that influence predictions. By making model behavior more understandable, XAI helps reduce bias, increase trust, and improve the overall reliability of AI systems.^28^, ^29^ A widely used XAI technique is Gradient-weighted Class Activation Mapping (Grad-CAM). Grad-CAM generates visual explanations by highlighting the image regions most relevant to the model’s predictions.^30^ This method uses the gradients of the target class flowing into the final convolutional layer of a neural network to produce class-discriminative heat maps. In this study, we applied Grad-CAM to interpret the model’s behavior during testing across all cross-validation folds.

## Results and evaluation

We conducted two primary experiments, detailed in the Methods section. In Experiment 1, we trained a classification model to distinguish between chest compression signals and ROSC. Among the evaluated models, VGG16 achieved the highest performance, surpassing other SOTA models in accurately distinguishing these two classes. Other models failed to reliably differentiate the signal types.

In Experiment 2, we trained a two-stage classifier. In the first stage, we differentiated between compressions and ROSC, similar to Experiment 1. In the second stage, we further classified compression signals into compressions and compressions with intrinsic cardiac activity. Among the models tested, VGG16 outperformed other SOTA models. Although MobileNet also performed well, it tended to identify noisy regions as vital biomarkers for classification. Quantitative analysis of VGG16 yielded a combined mean accuracy of 98 %, sensitivity of 98 %, specificity of 97 %, PPV of 97 %, and NPV of 100 % across a two-fold cross-validation process. Evaluation metrics were estimated using a weighted average method. In stage 1, the VGG16 model achieved mean sensitivity and specificity of 98 % each, whereas, in stage 2, it achieved mean sensitivity and specificity of 99 % and 96 %, respectively. These quantitative evaluations demonstrate that the two-stage detector model is capable of identifying ROSC with a sensitivity of 98 % in stage 1 and detecting compressions with intrinsic cardiac activity with a sensitivity of 99 % in stage 2.

**Fig. 5:**
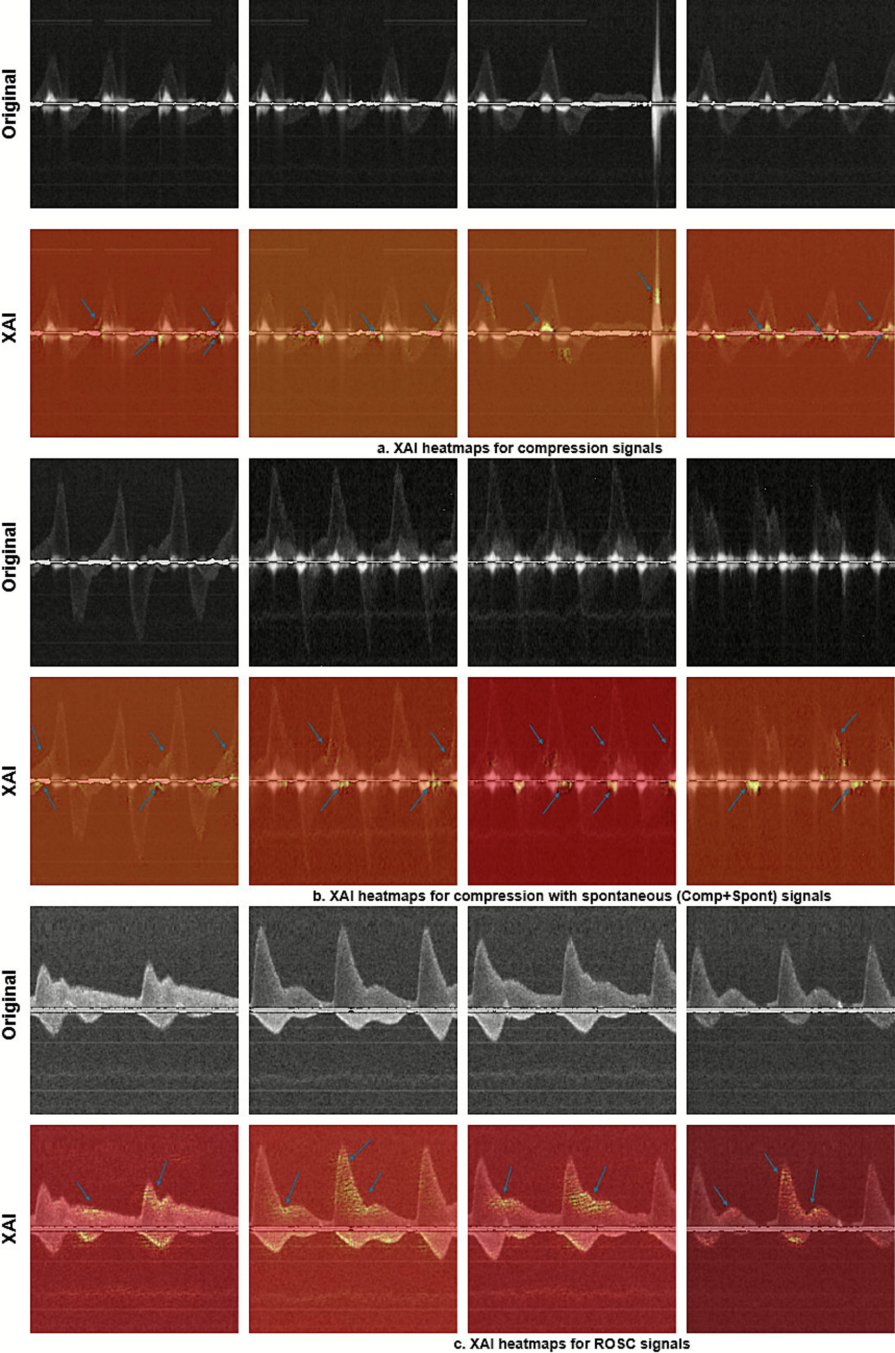
**Explainable AI heatmaps for predictions of VGG16. (a) represents the biomarkers corresponding to compression signals, (b) the biomarkers corresponding to compressions with intrinsic cardiac activity and (c) the biomarkers corresponding to ROSC signals. Regions of interest are pointed out using arrow marks**.

Fig. 5 presents the qualitative evaluation of our model’s performance using Grad-CAM to identify significant features influencing predictions. In the Grad-CAM heatmaps of compression signals (Fig. 5a), we observed that the model focuses on important features such as positive blood flow regions and baseline noise. For compressions involving intrinsic cardiac activity signals (Fig. 5b), key features include the positive velocity peak of compressions and intrinsic cardiac activity peaks. In the case of ROSC signals (Fig. 5c), the presence of diastolic flow and dicrotic notch regions are the primary influences on the model’s predictions. The clinical relevance of vital biomarkers highlighted in the XAI visualizations has been validated by experienced personnel. Additionally, the model effectively identifies key features even in low-intensity signals (Fig. 5a and b).

## Discussion

This study demonstrates the feasibility of using deep learning for real-time detection of ROSC and intrinsic cardiac activity during chest compressions. This is based on carotid blood flow velocities in an animal model. In cases of cardiac arrest, it is crucial to determine whether the patient has an active circulation and if chest compressions are effectively delivering blood to the brain. Both these factors are essential for optimizing resuscitation and improving outcomes. Currently, detection methods rely on manual pulse checks, which are unreliable and have only 54 % diagnostic accuracy for identifying arterial waveforms.^31^ Despite this, manual pulse checks remain the standard for identifying ROSC. Prompt recognition of ROSC allows rescuers to cease chest compressions, preventing unnecessary trauma and enabling a transition to post-resuscitation care.^32^, ^33^, ^34^ Delayed recognition can lead to over-compression, potentially causing harm or concealing signs of recovery. During CPR, chest compressions are the sole means of circulating blood to vital organs.

While RescueDoppler has demonstrated reliability during CPR,^6^ interpreting Doppler signals to ascertain the presence of intrisic cardiac activity during compressions and the onset of ROSC can be challenging for rescue personnel. Artificial intelligence has the potential to analyze complex physiological signals, such as Doppler ultrasound, to detect ROSC. AI could provide consistent, data-driven insights, thereby reducing the risk of errors or missing signs of recovery.

Several studies have investigated AI-assisted tools for managing cardiac arrest using ECG, capnography, and Doppler data.^17^, ^35^, ^19^, ^36^, ^37^, ^38^, ^39^ However, research involving AI in porcine models remains limited.^40^, ^41^, ^42^, ^43^ Suresh et al.^41^ employed statistical methods to evaluate whether regional cerebral oximetry (*rSO_2_*) during CPR could predict ROSC and neurological outcomes. Despite this effort, oximetry is often unreliable due to interference^44^ and the absence of standardized thresholds.^45^

Kim et al.^40^ compared AI-driven robots with the mechanical compression device LUCAS3, finding no significant differences in performance with a porcine model of cardiac arrest. However, their study did not include manual CPR as a comparison. Reynolds et al.^42^ applied linear models to NIRS data but highlighted the models’ inability to distinguish between hemoglobin and myoglobin, thus limiting reliability. Jiang et al.^43^ identified coronary perfusion pressure (CPP) as a strong indicator of ROSC. However, they relied on ECG and PPG data, which do not provide real-time blood flow insights. For data analysis, they employed simple machine learning models, including support vector machines (SVM), k-nearest neighbors (KNN), and tree-based algorithms.

To our knowledge, no studies have specifically examined the application of AI in carotid ultrasound Doppler imaging for distinguishing between signals of chest compression, compressions with intrinsic cardiac activity, and ROSC. To address these challenges, we conducted a retrospective study using porcine data and advanced deep learning models.

Cherry et al.^46^ observed that the physiology of pigs closely resembles that of humans, rendering porcine cardiac arrest models clinically relevant. Key biomarkers for confirming the ROSC include a mean arterial pressure of ≥65 mmHg,^47^, ^48^ peripheral oxygen saturation (SpO2) of 94–98 %,^49^ and diastolic flow,^50^ which indicate sufficient coronary perfusion pressure during resuscitation. In our controlled study, as confirmed by Faldaas et al., ROSC was successfully achieved, with all biomarkers carefully monitored.

We identified that the presence of diastolic flow serves as a vital biomarker for classifying the given signals, as indicated visually by heatmaps in Fig. 5. Clinicians validated these biomarkers to confirm their clinical relevance. The model recognized both diastolic flow and dicrotic notch regions as key biomarkers of the ROSC signal, emphasizing their clinical importance. The presence of diastolic flow suggests adequate oxygenation and blood supply. Furthermore, the model identified positive flow patterns and baseline noise as crucial biomarkers of chest compression. In signals comprising intrinsic cardiac activity during compressions, the model identified positive peaks of intrinsic activity, compression peaks, and baseline noise as essential biomarkers.

Chest compressions create motion artifacts, visible as noise along the baseline in the B-mode spectrum (Fig. 3, Fig. 5). Negative blood flow velocities, which indicate retrograde blood flow, are commonly observed during compressions.^51^ Although rescue personnel are aware of performing compressions, it remains essential to identify intrinsic cardiac activity during compressions to mitigate the negative effects of prolonged compressions. Our two-stage detector effectively identified these signals with high sensitivity and specificity.

During the evaluation, we measured inference speed and found that the model can process 180 images per second. Additionally, we tested our models on the CPU and observed an inference speed of 114 images per second, demonstrating that inference does not require a modern GPU setup to achieve real-time performance.

### Limitations and future work

Although our results are promising, several key challenges persist. The use of an animal model in a controlled setting limits direct applicability to humans, emphasizing the need for clinical validation, which remains a primary focus of our work. A pilot study of cardiac arrest on humans has been conducted by Krüger et. al and as part of our future efforts, we will conduct experiments using data obtained from human trials to rigorously validate the performance and feasibility of real-time deployment of AI methods in real clinical environments. Although we aim to develop more accurate, real-time deep learning models, the limitations of current data underscore the necessity of expanding to larger, well-annotated datasets. Such expansion is crucial to enhancing robustness and reliability across diverse clinical scenarios. Even though the models demonstrated real-time inference speeds during tests on both GPUs and CPUs, further validation using data from clinical trials is necessary to confirm the feasibility of real-time implementation.

Another significant limitation is that the current models are not trained to recognize asystole, pulseless electrical activity or ventricular fibrillation, This restriction reduces the models’ diagnostic scope and underscores the necessity for broader rhythm classification in future iterations. Artificial intelligence offers the potential to integrate multiple physiological modalities, including ECG, capnography, and Doppler ultrasound, to detect subtle patterns indicative of effective circulation. Such multimodal integration could improve prediction accuracy for outcomes such as ROSC and support hemodynamically guided, personalized resuscitation strategies.

## Conclusions

AI-enhanced carotid blood flow monitoring during CPR demonstrates strong potential for real-time detection of ROSC and intrinsic cardiac activity during chest compressions in an animal model. This approach overcomes the limitations of manual pulse checks. Utilizing a two-step methodology, the VGG16 model achieved 98 % accuracy. Additionally, XAI heatmaps highlighted key features, such as flow patterns, compression peaks, intrinsic activity, and diastolic flow during ROSC.

## Declaration of Generative AI and AI-assisted technologies in the writing process

ChatGPT has been used for language correction and to improve the readability of manuscript. No other tools have been used for writing this manuscript, and we take full responsibility for the content in the manuscript.

## CRediT authorship contribution statement

**Raghava Vinaykanth Mushunuri:** Writing – original draft, Visualization, Software, Methodology, Investigation, Formal analysis, Conceptualization. **Bjorn Ove Faldaas:** Writing – review & editing, Data curation, Conceptualization. **Frank Lindseth:** Writing – review & editing, Validation, Methodology, Investigation. **Charlotte Bjork Ingul:** Writing – review & editing, Methodology, Investigation, Formal analysis, Conceptualization. **Gabriel Kiss:** Writing – review & editing, Validation, Supervision, Software, Methodology, Conceptualization.

## Declaration of competing interest

Charlotte Björk Ingul serves as a Medical Advisor at Cimon Medical. All other authors have no conflicts of interest.
