# Supplementary material for: Detection of return of spontaneous circulation during cardiopulmonary resuscitation using continuous carotid artery Doppler blood flow monitored by AI in an animal model
Source: Resusc Plus. 2025 Dec 24;27:101207. doi: 10.1016/j.resplu.2025.101207 (PMC12814833; doi:10.1016/j.resplu.2025.101207)
Supplement: Supplementary Data 1 [file mmc1.docx]

**The ARRIVE Guidelines**

**Animal Research: Reporting *In Vivo* Experiments**

|  | **Item** | **Recommendation** |
| --- | --- | --- |
| **TITLE:Detection of Return of Spontaneous Circulation during**  **Cardiopulmonary Resuscitation using Continuous Carotid Artery**  **Doppler Blood Flow monitored by AI in an Animal model of**  **Cardiac Arrest** |  |  |
| **ABSTRACT**  Manual pulse palpation during cardiopulmonary resuscitation (CPR) to check for spontaneous circulation is often unreliable and time-consuming. To address this, a novel RescueDoppler device has been developed, consisting of a small ultrasound probe that attaches to the neck and continuously monitors potential blood flow in the carotid artery. To assist rescuers, automatic real-time feedback on the return of spontaneous circulation (ROSC) is needed. |  |  |
| **INTRODUCTION** |  |  |
| - **Background:**   RescueDoppler is able to provide blood flow information during CPR, however interpreting the signals requires experienced personnel. AI has the potential to provide distinguish various signal types. Therefore, we trained state-of-the-art AI models to identify the presence of ROSC and spontaneous circulation during compressions. |  |  |
| - **Objectives:** To provide automatic feedback on ROSC and spontaneous circulation during compressions using RescueDoppler carotid blood flow during cardiac arrest by employing advanced deep-learning techniques. |  |  |
| **METHODS** |  |  |
| - **Ethical statement:** We state that this study has been conducted according to the ARRIVE guidelines. Animal welfare is the top priority for the research team |  |  |
| - **Study design:** This study has been designed to evaluate the performance of state-of-the-art Deep learning models in identifying ROSC during CPR using RescuDoppler carotid bloodflow signals. |  |  |
| - **Experimental procedures:**   We conducted a restrospective study using the recordings where ventricular fibrillation was induced via implantable cardioverter-defibrillator. Annotation tool has been designed to annotater the heart cycles. Deep learning models are trained with one second and two second spectrum signals and explainable AI methods have been employed to observe the vital features which contributed the most for model’s prediction |  |  |
| - **Experimental animals**   Pigs (Sus scrofa domesticus). |  |  |
| - **Housing and husbandry**   All animals were retrieved from a local specific pathogen-free (SPF) certified farm. |  |  |
| - **Sample size**   We used recordings of 9 animals. |  |  |
| - **Allocating animals to experimental groups**   Not relevant. |  |  |
| - **Experimental outcomes:**   Deep learning model to detect ROSC and spontaneous circulation during compressions |  |  |
| - **Statistical methods**   Sensitivity, specificity, Negative predictive value (NPV) and positive predictive value (PPV) have been evaluated to analyse the performance of the model quantitatively. XAI heatmaps have been analysed qualitatively. |  |  |
| **RESULTS** |  |  |
| - **Baseline data**   Two stage classification Model with sensitivity of 97% and specificity of 95% have been achieved for identification of ROSC and compressions with spontaneous circulation. |  |  |
| - **Numbers analyzed**   Sensitivity, specificity, NPV and PPV |  |  |
| - **Outcomes and estimation**   We aimed to evaluate if deep learning models can identify ROSC and compressions with spontaneous circulation. |  |  |
| - **Adverse events**   None |  |  |
| **DISCUSSION** |  |  |
| - **Interpretation/scientific implications.**   Data from RescueDoppler device is analysed to distinguish ROSC and compressions with spontaneous circulation. We observed that dicrotic notch and diastolic is key feature for identifying ROSC signals and spontaneous peaks, compression peaks as well baseline noise are vital for distinguishing compressions with spontaneous circulation signals. |  |  |
| - **Generalisability/translation**   We aim to transcend the research from Animal study to human data using transfer learning approach, to study how well the knowledge of AI models can be employed across the domains. |  |  |
| - **Funding**   Norwegian Research Council  Liaison Committee for Central Norway Regional Health Authority and the Norwegian University of Science and Technology |  |  |
